# Study on mitogenic activity of serum from patients with total coronary occlusions: relation to duration of occlusion

**DOI:** 10.1007/s10456-024-09958-0

**Published:** 2024-12-02

**Authors:** V. Tchaikovski, G. S. Werner, M. Fritzenwanger, E. Jandt, Johannes Waltenberger

**Affiliations:** 1 Department of Cardiology, Cardiovascular Research Institute Maastricht (CARIM), Maastricht University Hospital, Maastricht University, Maastricht, The Netherlands; 2 Clinic for Internal Medicine I, Friedrich-Schiller-University Jena, Jena, Germany; 3 Department of Cardiology, Angiology, Pulmonology, Nephrology and Intensive Care, Brandenburg University Hospital, Brandenburg an der Havel, Germany; 4 Department of Cardiovascular Medicine, Medical Faculty, University of Muenster, Albert-Schweitzer-Campus 1 – A1, 48149 Münster, Germany; 5 Cells-in-Motion Cluster of Excellence (EXC 1003—CiM), University of Münster, Münster, Germany; 6 Hirslanden Clinic in Park, Cardiology, Zurich, Switzerland

**Keywords:** Atherosclerosis, Coronary collaterals, Cell function, Growth factors

## Abstract

In contrast to the extensive evidence from animal studies, only few human data are available on the relation of vascular growth factors and collateral function as well as on the conditions which may modify their release or function. In 31 patients with total coronary occlusion (TCOs) blood was collected from distal to the occlusion site (collateral circulation) and from the aortic root (systemic circulation). Serum was used to assess its mitogenic potential in [^3^H]-thymidine incorporation assay on human umbilical vein endothelial cells. Serum from patients with the duration of occlusion between 1 and 3 months was significantly more mitogenic as compared to either shorter or longer duration of occlusion. None of the demographic or clinical factors correlated with the mitogenic activity of serum. Serum from patients with TCOs shows a particular time-dependent mitogenic profile with a maximal activity between 1 and 3 months following the occlusion. This profile corresponds to the experimentally described time-line of strongest collateral development and indicates the time-window for possible modification.

Dear Editor,

The collateral circulation of the heart has the potential to significantly change the natural course of coronary artery disease due to its enormous adaptive power. Collateral vessels can, upon demand, expand their diameter and flow capacity in experimental [1] and clinical [2] settings. Patients responding with an adequate collateral development present with minimal symptoms in settings of acute myocardial ischemia [2]. The process of collateral development/maturation following the occlusion of the major coronary artery is orchestrated by (a) re-directed blood flow and therefore significantly increased shear stress [1], (b) elevated production of cyto- and chemokines triggered by myocardial ischemia and released locally in ischemic myocardium leading to the maturation of preexisting interarterial connections [1].

Despite extensive preclinical evidence [2], there are only few data demonstrating a functional role of these factors in the development of collateral arteries in humans. Elevated levels of predominantly basic fibroblast growth factor (bFGF) were shown in patients with chronic total coronary occlusions (TCOs) [3]. A further understanding of their role in collateral development in humans may potentially impact the decision making in type and time of therapeutic interventions in ischemic heart disease [2].

Among others proliferation of the luminal endothelial cells plays a crucial step in the process of collateral maturation. In the present study we have investigated the total proliferative activity of serum from the collateral circulation (CC) on endothelial cells and compared these data with the proliferative activity of serum from the systemic circulation (SC) of the same patients with TCOs. In 31 consecutive TCO patients, blood was collected distal to the occlusion (collateral circulation, CC) and from the aortic root (systemic circulation, SC) just prior to revascularization [3]. The study protocol had been approved by the ethics committee of Jena University (Germany), and written informed consent was obtained; the blood samples were processed as described [3]. The inclusion criteria were (1) duration of the occlusion > 2 weeks on the basis of a previous angiogram, the date of a prior myocardial infarction, or the onset of symptoms; (2) TIMI 0 coronary flow; and (3) evidence of ischemia related to the occlusion or viable myocardium in case of regional akinesia [3]. The mitogenic activity of serum was assessed using human umbilical vein endothelial cells (HUVEC) in vitro and a [^3^H]-thymidine incorporation-based proliferation assay, which uses DNA synthesis as a marker of the proliferative cellular activity [4]. For the experiments we used the 10% serum concentration (data not shown and [4]).

The population characteristics are presented in the Table 1. The corresponding groups did not differ from each other with regard to clinical parameters. Serum from patients with the duration of occlusion between 1 and 3 months triggered significantly higher DNA synthesis (*p* < 0.05) as compared to both the samples from occlusions of either shorter or longer duration (Figure). The presence of DM, different NYHA classes, previous myocardial infarction, regional myocardial function, demographic parameters did not correlate with the mitogenic activity of the serum (data not shown). Serum from CC did not exhibit a higher mitogenic potential as compared to SC (Figure 1).

**Fig. 1:**
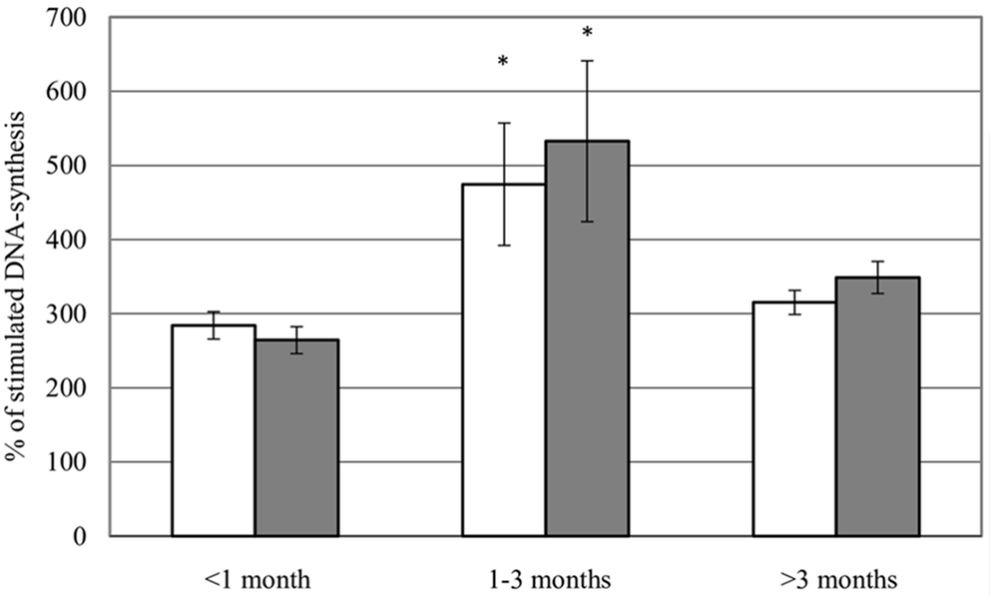
Proliferative activity of serum from patients with TCO, data are presented as mean ± standard error of mean; open bars – serum from CC, grey bars – serum from SC; * - *p* < 0.05 as compared to serum from either group (“<1 month” or “>3 months”)

**Table 1:**
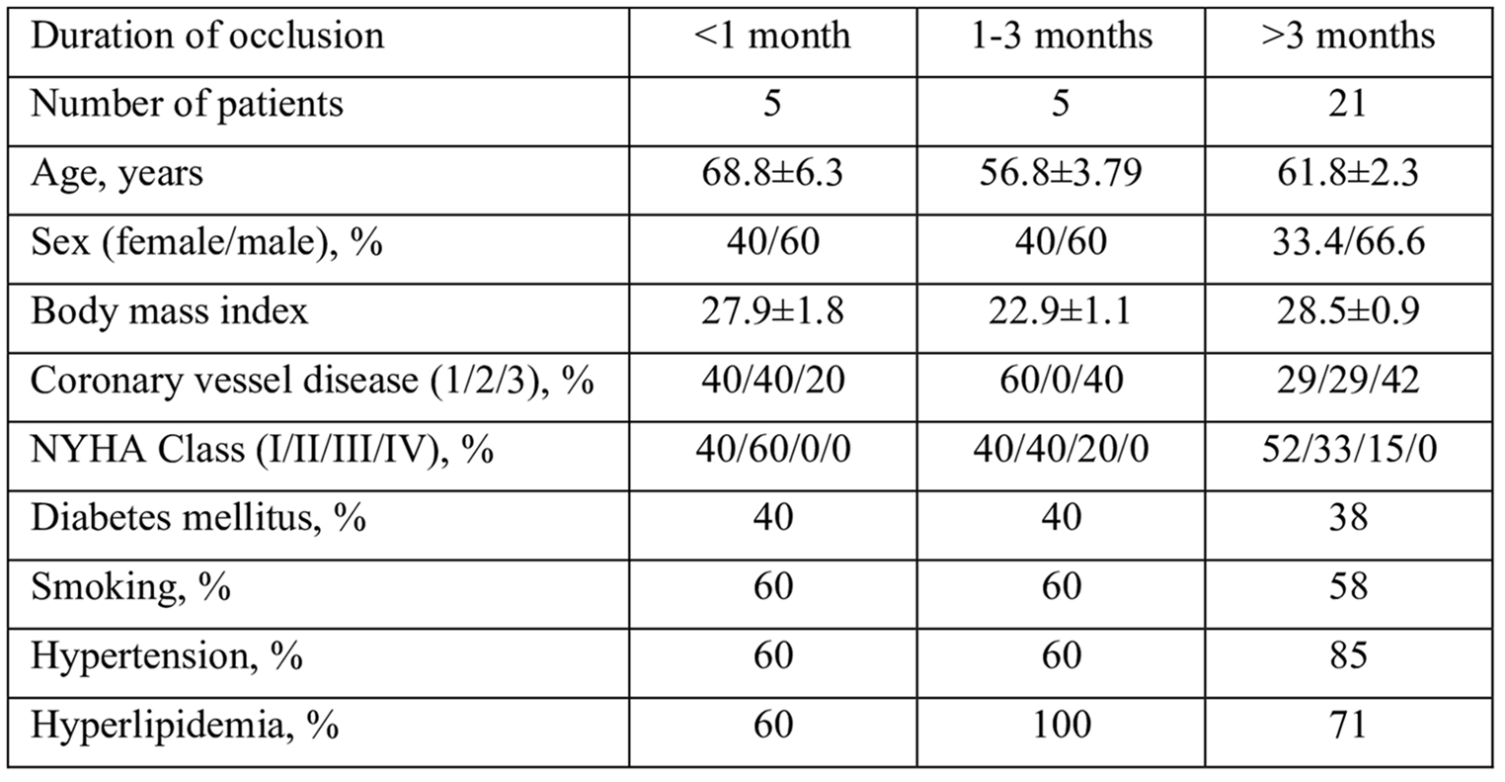
Study population characteristics, where appropriate data are presented as mean ± standard error of mean

Our major finding was that the proliferative potential of serum is maximal in the period between 1 and 3 months following a complete occlusion of a major coronary artery. This observation reflects the situation in large animals, where expression of vascular proteins involved in the growth of coronary collateral arteries returned to almost normal at 8 weeks after induction indicating cessation of remodeling [1]. Therefore our data confirm the use of large animal models to study the process of collateral development and arteriogenesis. Moreover, our data confirm the findings on the development of collaterals as assessed angiographically and functionally in human TCOs [2].

Furthermore, the description of this particular time window identifies opportunities for therapeutic interventions in situations, where collateral formation is either altered or delayed. There is experimental evidence showing that the expression of growth factors may be altered in DM [1] and that the cellular response is impaired [5, 6]. It has been shown that patients with diabetes mellitus (DM) have reduced number of collaterals [7] and reduced collateral function [8]. In the current study, however, we did not see any difference in the proliferative activity of the sera between patients with and without DM. This further supports the hypothesis that the defect in DM lays - most likely - at the cellular level [6, 9].

Serum from CC did not exhibit a higher mitogenic potential as compared to SC. This is probably due to a wash-out effect. Higher levels of bFGF have been reported in the coronary sinus of patients without TCOs but not in patients with TCOs, however the difference was only 2-fold and most likely not sufficient to cause a significant proliferative response [3]. We therefore suppose that intra-tissue concentrations of the corresponding growth factors are stronger elevated than in the serum.

Serum from patients with TCO shows a time-dependent mitogenic trend with a maximal activity between 1 and 3 months following the occlusion (Figure). These data support a crucial role of growth factors in the development of collateral circulation and provide further insight into the dynamics of the process of collateral development. These findings should further support the development of interventional strategies for therapeutic arteriogenesis.

## Data Availability

No datasets were generated or analysed during the current study.
